# Analysis of Signaling Endosome Composition and Dynamics Using SILAC in Embryonic Stem Cell-Derived Neurons*[Fn FN2]

**DOI:** 10.1074/mcp.M115.051649

**Published:** 2016-02

**Authors:** Solène Debaisieux, Vesela Encheva, Probir Chakravarty, Ambrosius P. Snijders, Giampietro Schiavo

**Affiliations:** From the ‡Molecular NeuroPathobiology Laboratory, Sobell Department of Motor Neuroscience and Movement Disorders, UCL Institute of Neurology, University College London, London WC1N 3BG, UK;; §Bioinformatics and Biostatistics Group, The Francis Crick Institute, London WC2A 3LY, UK;; ¶Protein Analysis and Proteomics Group, The Francis Crick Institute, South Mimms EN6 3LD, UK

## Abstract

Neurons require efficient transport mechanisms such as fast axonal transport to ensure neuronal homeostasis and survival. Neurotrophins and their receptors are conveyed via fast axonal retrograde transport of signaling endosomes to the soma, where they elicit transcriptional responses. Despite the essential roles of signaling endosomes in neuronal differentiation and survival, little is known about their molecular identity, dynamics, and regulation. Gaining a better mechanistic understanding of these organelles and their kinetics is crucial, given the growing evidence linking vesicular trafficking deficits to neurodegeneration. Here, we exploited an affinity purification strategy using the binding fragment of tetanus neurotoxin (H_C_T) conjugated to monocrystalline iron oxide nanoparticles (MIONs), which in motor neurons, is transported in the same carriers as neurotrophins and their receptors. To quantitatively assess the molecular composition of H_C_T-containing signaling endosomes, we have developed a protocol for triple Stable Isotope Labeling with Amino acids in Cell culture (SILAC) in embryonic stem cell-derived motor neurons. After H_C_T internalization, retrograde carriers were magnetically isolated at different time points and subjected to mass-spectrometry and Gene Ontology analyses. This purification strategy is highly specific, as confirmed by the presence of essential regulators of fast axonal transport in the make-up of these organelles. Our results indicate that signaling endosomes undergo a rapid maturation with the acquisition of late endosome markers following a specific time-dependent kinetics. Strikingly, signaling endosomes are specifically enriched in proteins known to be involved in neurodegenerative diseases and neuroinfection. Moreover, we highlighted the presence of novel components, whose precise temporal recruitment on signaling endosomes might be essential for proper sorting and/or transport of these organelles. This study provides the first quantitative proteomic analysis of signaling endosomes isolated from motor neurons and allows the assembly of a functional map of these axonal carriers involved in long-range neuronal signaling.

Intracellular communication is essential to maintain neuronal homeostasis, differentiation and survival. Motor neurons (MNs)^1^ are characterized by very long axons whose nerve terminals can be as far as one meter away from the soma in humans. These long distances require the development of specialized mechanisms to ensure effective long-range communication between axonal and somatodendritic compartments. Fast axonal transport represents the backbone of these trafficking mechanisms and is responsible for the shuttling of several types of organelles, proteins, and RNAs on microtubule tracks (1, 2). Anterograde transport is mainly driven by kinesin motors and ensures delivery of newly synthesized proteins, synaptic vesicle precursors, lipids, and organelles to synapses and distal parts of dendrites. In contrast, retrograde transport relies on cytoplasmic dynein for the movement of mitochondria, autophagosomes, lysosomes, and aging proteins targeted for degradation and/or recycling from axon terminals to the soma. Other cargoes such as neurotrophins (NTs) and their receptors are transported in a retrograde fashion. Upon binding to their receptors, NTs are internalized in endocytic carriers and transported along the axon to the soma, where they exert their trophic responses (3, 4). These specialized organelles, the so-called signaling endosomes, are key players in this process. On their delimiting membrane, signaling endosomes host several factors required for NT signaling (2) and for their sorting and transport, such as the small GTPases Rab5 and Rab7 (5, 6) (Fig. 1*A*).

Increasing evidence show that deficits in axonal transport lead to neurodevelopmental and neurodegenerative diseases. Mutations in molecular motors, their regulators, and cytoskeletal components have been associated with motor neuron disease, Alzheimer's disease and Huntington's disease, to cite a few (7, 8). Moreover, mutations in Rab proteins and their regulating factors cause motor and sensory pathologies, such as Charcot-Marie-Tooth type 2B (CMT2B) disease, which is caused by mutations in Rab7 (9) and a familial form of amyotrophic lateral sclerosis (ALS), which is linked to mutations in the Rab5 regulator alsin (10). Further links between motor neuron disease and mutations of the dynein/dynactin complex have also been reported (2, 11, 12).

Although several studies focused on unraveling the signaling properties, the role of actin and microtubules-dependent motors, and the sequential recruitment of Rab5 and Rab7-GTPases on signaling endosomes (13) (Fig. 1*A*), little is known about their molecular dynamics and temporal changes in their composition during transport. Different approaches have been pursued to investigate the composition of signaling endosomes in neurons, ranging from the use of a sciatic chamber (14), to subcellular fractionation and pull-down (15), immunoisolation (16), high-resolution organelle fractionation (17), and, more recently, ferrofluid-mediated magnetic purification (18). Those studies yielded new data regarding the composition of signaling endosomes, yet a quantitative proteomic approach would describe better the nature of those endocytic and provide more details about their dynamics and molecular make-up (19). Various mass spectrometry (MS) strategies have been applied to study the molecular composition of purified synaptic vesicles (20) and synapses (21, 22), NT signaling pathways (23), and to evaluate the functional differences between primary MNs and MN-like cell lines (24). Considering the established link between axonal transport deficits and amyotrophic lateral sclerosis, a neurodegenerative diseases affecting MNs (25), this type of neurons constitute a model of choice to investigate the molecular composition of signaling endosomes and its modifications during axonal transport. Temporal changes in the proteome can be measured by Stable Isotope Labeling with Amino acids in Cell culture (SILAC), a quantitative method involving metabolic labeling, which allows the assessment of the relative abundance of proteins and their changes in different experimental conditions. Given their terminal differentiation, primary MNs have low protein turnover rates, making it difficult to achieve complete metabolic incorporation (26). We circumvented this difficulty by developing SILAC protocols using mouse embryonic stem (ES) cell-derived MNs, in which labeled amino acid incorporation was set up from the pluripotent stem cell stage, allowing efficient labeling of the generated neurons. As described previously (6, 27, 28), we exploited the ability of the binding fragment of tetanus neurotoxin (H_C_T) to be internalized in MNs and undergo axonal retrograde transport to perform pulse-chase experiments and purify H_C_T-containing endosomes from SILAC-labeled cells. Gene Ontology (GO) analysis showed that the signaling endosomes underwent a fast maturation process, mediated by a progressive enrichment of late endosome (LE)/lysosome markers. In addition, our results indicated that several proteins reported to be involved in pathologies affecting the nervous system are associated to signaling endosomes, emphasizing the pathophysiological relevance of our study. Importantly, our data also revealed potential new signaling endosome components, whose temporal recruitment and/or function might be essential for axonal retrograde transport.

## EXPERIMENTAL PROCEDURES

### 

#### 

##### Cells and Reagents

Mouse ES cells were provided by the Crick Institute Biological Resource Unit and were derived from hybrid blastocysts generated by the mating of C57BL/6J and 129 (S6)SvEv mice as previously described (29).

Unlabeled l-arginine and l-lysine, (R0K0, Sigma-Aldrich, Gillingham, United Kingdom), medium l-arginine [^13^C_6_] and l-lysine [D_4_] R6K4) and heavy l-arginine [^13^C_6,_^15^N_4_] and l-lysine [^13^C_6,_^15^N_2_] (R10K8) in their hydrochloride forms were obtained from CK Isotopes (Ibstock, United Kingdom).

Rat antihemagglutinin (HA; Roche, Burgess Hill, West Sussex, United Kingdom; 1:1000), rabbit anti-Arl8b (Biorbyt, Cambridge, United Kingdom; 1:200), goat anti-LIMP2 and anti-CAR (R&D Systems, Abington, United Kingdom; both 1:100) primary antibodies were used for immunofluorescence. For western blots, we used the following: mouse anti-Rab5 (Synaptic Systems, Göttingen, Germany; 1:500), rabbit anti-Rab7 (Cell Signaling Technology, Leiden, The Netherlands; 1:1000), rabbit anti-Arl8b (1:500) (30), goat anti-CAR (R&D Systems, 1:500) and mouse anti-βIII-tubulin (Covance, London, United Kingdom; 1:1000).

##### Cell Culture, Differentiation, and Metabolic Labeling

ES cells were grown for 4 days on 0.2% fish skin gelatin-coated flasks in SILAC growth medium made of custom-made SILAC Dulbecco's Modified Eagle Medium:nutrient mixture F12 (DMEM/F12, AthenaES-Enzo Life Sciences, Newmarket, United Kingdom) supplemented with light, medium or heavy l-Arg (147.5 mg/L) and l-Lys (91.25 mg/L), 10% KnockOut™ serum replacement (KOSR; Life Technologies, Paisley, United Kingdom), 1% GlutaMAX™ (Life Technologies), 0.1 mm 2-mercaptoethanol, and 1000 units/ml of ESGRO® recombinant leukemia inhibitory factor (Millipore, Watford, United Kingdom). To generate MNs, SILAC-ES cells were transferred to nontissue culture-treated Petri dishes and grown in suspension in differentiation medium composed of 45% custom-made SILAC Neurobasal® medium (Cell Services, Crick Institute) supplemented with corresponding types of l-Arg (84 mg/L) and l-Lys (146 mg/L), 45% SILAC DMEM/F12 supplemented with 147.5 mg/L of l-Arg and 91.25 mg/L of l-Lys, 10% KOSR, 0.4% GlutaMAX and 0.1 mm 2-mercaptoethanol (day 0 of differentiation). At day 1, embryoid bodies (EBs) were gently centrifuged, resuspended in fresh SILAC differentiation medium and transferred to new Petri dishes. The following day (day 2), the enlarged EBs were allowed to sediment by gravity before being re-suspended in fresh SILAC differentiation medium supplemented with 1 μm retinoic acid (RA; Sigma-Aldrich) and 333 nm Smoothened ligand SAG, a chemical activator of sonic hedgehog signaling (Enzo Life Sciences). EBs were maintained under these conditions for 4 days with medium changed every day and then gently disaggregated at day 7 with Accumax™ solution (Millipore). Dissociated cells were then plated onto poly-d,l-ornithine and laminin (Sigma-Aldrich) coated dishes and/or glass coverslips in SILAC MN growth medium made of 95% supplemented SILAC Neurobasal® medium, completed with 2% B27, 2% heat-inactivated dialyzed horse serum (1 kDa MWCO, Dundee Cell Products, Dundee, United Kingdom), 1% GlutaMAX, 25 μm 2-mercaptoethanol, 10 ng/ml rat ciliary neurotrophic factor (R&D Systems), 0.1 ng/ml rat glial cell line-derived neurotrophic factor (R&D Systems), and 1 μm RA. Cells were maintained during 3 days in culture (DIV3) before use.

##### Signaling Endosome Purification

Amino-derivatized monocrystalline iron oxide nanoparticles (MIONs, Kisker, Steinfurt, Germany) were conjugated to cysteine-rich HA-tagged H_C_T or glutathione-S-transferase (GST) as previously described (6, 27). Briefly, 5 mg of MIONs were activated with 1 mm EDTA and 3 mm succinimidyl 4-(N-maleimidomethyl)cyclohexane-1-carboxylate (Life Technologies) for 30 min at 22 °C. After having repeated this step once, activated MIONs were purified on a PD10 desalting column (GE Healthcare, Little Chalfont, United Kingdom), concentrated and divided into two batches, which were labeled for 2 days at 4 °C with 6 nmol of H_C_T or GST, both previously reduced with 1 mm Tris(2-carboxyethyl)phosphine (TCEP; Pierce, Paisley, United Kingdom) for 30 min at 22 °C. The reaction was blocked by incubation with 2 mm reduced glutathione (Sigma-Aldrich) for 1 h at 22 °C. Conjugated MIONs were purified on Sephacryl S100HR (GE Healthcare).

The purification of MNs signaling endosomes was performed as previously described (6, 27) with minor modifications. H_C_T-MIONs and GST-MIONs were incubated with DIV3 ES-derived MN for 60 min at 37 °C or pulsed for 10 min at 37 °C and then chased for 10, 30, and 60 min in complete MN medium. After internalization, MNs were cooled on ice, acid-washed, washed with Hank's balanced buffered salt solution (HBSS) pH 7.4 and finally with breaking buffer (BB, 0.25 m sucrose, 10 mm Hepes-KOH, pH 7.2, 1 mm EDTA, 1 mm magnesium acetate and protease inhibitors). Neurons were mechanically lysed in BB by passing them 15 times through a cell cracker device (18 μm clearance; European Molecular Biology Laboratory, Heidelberg, Germany) and clarified by centrifugation at 690 × *g* for 10 min at 4 °C (28).

For the SILAC experiment, the protein concentration of the three post-nuclear supernatants (PNS) was determined using a BCA protein quantification assay (Life Technologies), and the three PNS were mixed in 1:1:1 ratio. MS columns were placed inside a SuperMACS II (both from Miltenyi Biotech, Bisley, United Kingdom) and equilibrated with BB supplemented with 0.4% bovine serum albumin (BSA). The sample was then passed three times through the column. After several washes with BB, the columns were removed from the magnetic field and the elution was performed using 1 ml BB containing 300 mm KCl. Endosomal proteins were then precipitated and separated by SDS-PAGE using 10% Bis-Tris gels (Life Technologies).

##### In-gel Digestion and LC-MS/MS

Polyacrylamide gel slices were prepared for mass spectrometric analysis using the Janus liquid handling system (Perkin-Elmer, Seer Green, United Kingdom). Briefly, the excised protein gel piece was placed in a well of a 96-well microtiter plate and destained with 50% acetonitrile, 50 mm ammonium bicarbonate, reduced with 10 mm dithiothreitol, and alkylated with 55 mm iodoacetamide. Proteins were digested with 6 ng/μl trypsin overnight at 37 °C. The resulting peptides were extracted in 2% formic acid, 1% acetonitrile.

For LC-MS/MS analysis, peptides were loaded on 50 cm Easy Spray PepMap column (75 μm inner diameter, 2 μm particle size, Thermo Fisher, Paisley, United Kingdom) equipped with an integrated electrospray emitter. Reverse phase chromatography was performed using the RSLC nano U3000 (Thermo Fisher) with a binary buffer system at a flow rate of 250 nl/min. Solvent A was 0.1% formic acid, 5% dimethylsufoxide (DMSO), and solvent B was 80% acetonitrile, 0.1% formic acid, 5% DMSO. The in-gel digested samples were run on a linear gradient of solvent B (2–40%) in 65 min, total run time of 90 min including column conditioning. The Q Exactive was operated in data-dependent mode acquiring HCD MS/MS scans (*r* = 17,500) after an MS1 scan (*r* = 70,000) on the ten most abundant ions using MS1 target of 1 × 10^6^ ions, and MS2 target of 5 × 10^4^ ions. The maximum ion injection time utilized for MS2 scans was 0.120 s, the HCD normalized collision energy was set at 28, the dynamic exclusion was set at 10 s, and the peptide match and isotope exclusion functions were enabled.

##### MS Data Processing and Analysis

Raw data files were analyzed with MaxQuant/Andromeda software (version 1.3.0.5). Parent ion and tandem mass spectra were searched against *M. musculus* complete proteome containing 77,938 protein sequences obtained from UniprotKB (release August 2012). A list of 247 common laboratory contaminants provided by MaxQuant/Andromeda was also added to the database. The enzyme specificity was set to trypsin with a maximum of two missed cleavages. The precursor mass tolerance was set to 20 ppm for the first search (used for mass recalibration) and to 6 ppm for the main search. The tolerance for fragment ions was set at 20 ppm. Carbamidomethylation of cysteines was specified as fixed modification, oxidized methionines and N-terminal protein acetylation were searched as variable modifications. The datasets were filtered on posterior error probability (PEP) to achieve 1% false discovery rate on protein and peptide levels (56). Briefly, posterior error probabilities (PEP) were calculated for all peptide spectrum matches (PSMs; supplemental Table 1). The database containing all true protein sequences was concatenated with a decoy database containing reversed nonsense version of the sequences. The PEP scores were used to sort the PSM from forward and reverse databases until the desired FDR threshold is achieved (set at 1% for this study). The PEP value for a certain protein was calculated by multiplying their peptide PEPs and was used to sort the list of hits from forward and reverse databases. This specific SILAC experiment was performed once and the resulting raw data are available via the PRIDE database (http://www.ebi.ac.uk/pride/archive/, project accession: PXD002111).

##### Bioinformatics and Gene Ontology Analysis

To analyze the cell compartment distribution of SILAC proteins, all quantified proteins were annotated using *M. musculus* ontology gene sets downloaded from QuickGO (http://www.ebi.ac.uk/QuickGO/). The LE/lysosome gene set was obtained by merging LE and lysosome gene sets provided by QuickGO.

The enrichment of proteins (unique gene symbols) from the SILAC experiment was assessed using the “phyper” function in Bioconductor version 3 running R version 3.1.2. The function uses a hypergeometric test to evaluate the enrichment of proteins within the experiment relative to the mouse proteome for each GO set. All UniProtKB mouse identifiers mapped to unique gene symbols were used as the background. Disease and biological processes enrichment were assessed using a hypergeometric test, with the Metacore analysis software (http://www.Thompsonreuters.com). *p* values are multiple-testing corrected using the Benjamini-Hochberg method, where *p* < 0.05 are significant.

##### Western Blotting

After protein precipitation, purified proteins were separated by SDS-PAGE using NuPAGE Bis-Tris gradient gels (Life Technologies) and blotted overnight at 4 °C onto polyvinylidene fluoride membranes (GE Healthcare), according to the manufacturer's instructions. Membranes were blocked in 5% skimmed milk dissolved in Tris-buffered saline containing 0.05% Tween-20 (TBST) for 1 h at room temperature and then incubated with primary antibodies in TBST or TBST 5% skimmed milk overnight at 4 °C. Blots were then washed and incubated with appropriate horseradish peroxidase-conjugated secondary antibodies (GE Healthcare). Immunoreactivity was detected using enhanced chemiluminescence (Millipore and GE Healthcare).

##### Immunofluorescence and Confocal Microscopy

DIV3 ES-derived MNs plated on coverslips were incubated with HA-tagged H_C_T for 60 min at 37 °C or pulsed for 10 min at 37 °C, then chased for 10, 30, or 60 min in complete MN medium. After internalization, MNs were cooled on ice, acid-washed and washed with HBSS. Cells were then fixed in 4% paraformaldehyde in HBSS, quenched with 50 mm NH_4_Cl, pH 7.5, and permeabilized with 0.1% Triton X-100 in PBS. After blocking in PBS containing 5% BSA for 1 h at 25 °C, coverslips were incubated overnight at 4 °C with primary antibodies diluted in blocking buffer, washed and incubated with AlexaFluor-conjugated secondary antibodies (Life Technologies, 1:250 or 1:500) for 1 h at 25 °C. After several washes, coverslips were mounted in mowiol and confocal images were acquired using a Zeiss LSM780 equipped with a Plan-Apochromat 63x/1.40 oil DIC M27 objective. Images were then analyzed with Image J. The degree of colocalization between HA-H_C_T and other signaling endosome components was assessed by determining the Manders' coefficient using Image J. In the Arl8b validation experiment, the significance was assessed using one-way ANOVA (* < 0.05) followed by Dunnett's multiple comparisons test using the 10 min time point as reference.

## RESULTS

### 

#### 

##### H*_C_*T as an Effective Tool to Monitor Axonal Retrograde Transport of Signaling Endosomes

**Fig. 1.**
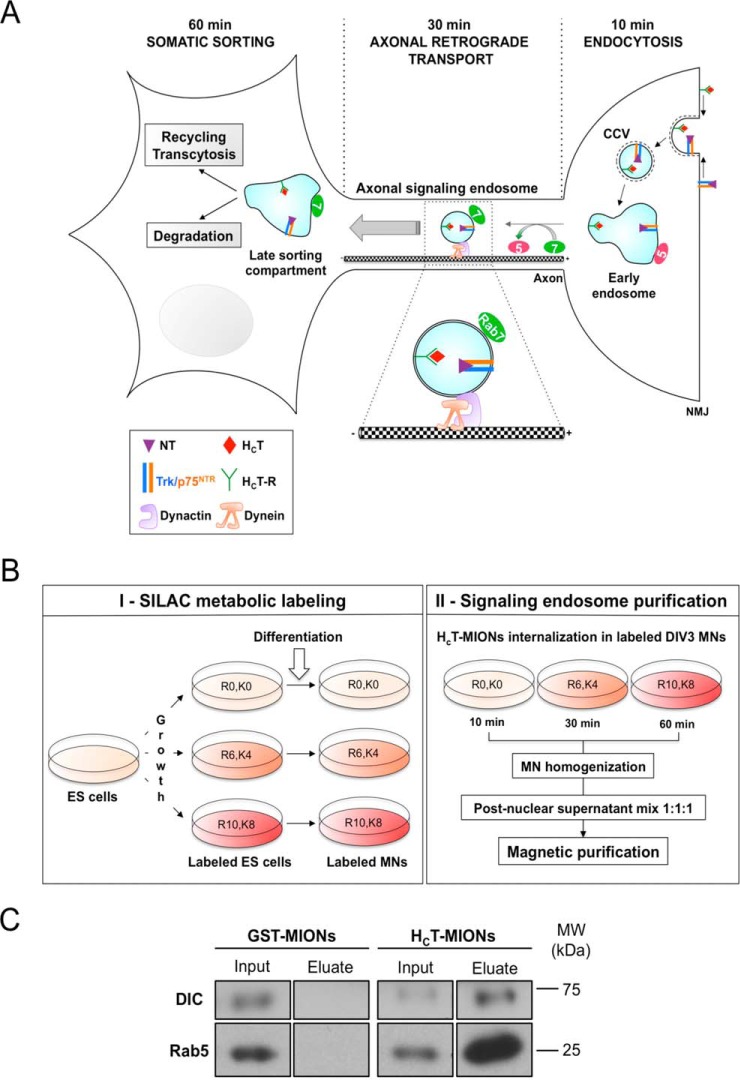
**Axonal transport of signaling endosomes and their analysis via SILAC.** *A,* Schematics summarizing current understanding on the trafficking of NTs, H_C_T and their receptors in MNs. At the neuromuscular junction (NMJ), H_C_T binds its plasma membrane receptors (71), whereas NTs bind Trks and p75^NTR^. Both complexes are rapidly internalized in clathrin-coated vesicles (CCV) before reaching a Rab5-positive early endosomal compartment characterized by slow short-range movements (10 min). Upon recruitment of Rab7 (30 min), signaling endosomes undergo microtubule-dependent fast axonal transport driven by cytoplasmic dynein. Once the soma is reached (60 min), receptor complexes are sorted to different fates: activated NT receptors are sent to degradation and/or recycled to the plasma membrane, whereas H_C_T complexes accumulate in perinuclear nonacidic compartments and/or undergo transcytosis to enter neighboring inhibitory interneurons. A full characterization of the somatic sorting compartment is still lacking. H_C_T: binding fragment of tetanus neurotoxin; H_C_T-R: H_C_T receptor complex; NT: neurotrophin, p75^NTR^; p75 neurotrophin receptor, Trk: tropomyosin receptor kinase; 5: Rab5; 7: Rab7. *B,* SILAC workflow. Label-free ES cells were split into three different ES cultures and each of them was incubated with light (R0K0), medium (R6K4) or heavy (R10K8) amino acids-supplemented growth medium for 4 days. ES cells were then subjected to an optimized MN differentiation protocol during which the labeled isotopes were maintained in the respective differentiation medias. At day 7 of differentiation, MNs were disaggregated, plated in MN medium supplemented with corresponding SILAC-labeled amino acids and maintained for 3 days (left panel, I). DIV3 MNs were pulsed for 10 min with H_C_T-conjugated MIONs and chased for 10, 30, or 60 min. MNs were then acid-washed, mechanically lysed, and cell homogenates were clarified by centrifugation. Postnuclear supernatants were pooled 1:1:1, and the resulting combined sample was submitted to magnetic purification protocol, as described under “Experimental Procedures” (right panel, II). *C,* Specificity of the purification strategy. DIV3 ES-derived MNs were incubated for 60 min at 37 °C with H_C_T- or GST-conjugated MIONs, acid-washed, and submitted to magnetic endosome purification. The inputs (post-nuclear supernatants, 3% of total) and the whole 60 min eluates were then separated by SDS-PAGE and analyzed by western blotting. MW: molecular weight, DIC, dynein intermediate chain.

Tetanus neurotoxin (TeNT) as well as its H_C_T binding fragment binds specifically to MNs at the neuromuscular junction, where it is internalized by clathrin-mediated endocytosis and retrogradely transported to the soma by axonal signaling endosomes. These organelles are characterized by long-range signaling activity, which enables specific transcriptional responses in the soma (13). Previous studies showed that H_C_T shares axonal transport carriers with NTs and their receptors in MNs and sensory neurons (31), making H_C_T a useful tool to visualize signaling endosomes and map their journey along the axon. At early time points after internalization, H_C_T-containing endosomes undergo slow short-range movements and are characterized by the presence of Rab5 on their limiting membrane (10 min; Fig. 1*A*). Rab5 is then exchanged with Rab7, a LE/lysosome marker, in a process that is required for fast axonal retrograde transport of these organelles to the soma (30 min; Fig. 1*A*) (6). Once they reached the soma, H_C_T and NT-receptor complexes are sorted to different fates: activated NT receptors are mainly sent to degradation (32), whereas H_C_T remains in nonacidic compartments (31, 33) and is transcytosed to adjacent interneurons (60 min; Fig. 1*A*). Although several studies used H_C_T as a probe to characterize retrograde carriers (6, 27, 28) little is known about the late sorting events occurring in signaling endosomes once they reach the soma, as well as the dynamic changes taking place during transport. Our study aims at unravel these mechanisms using H_C_T to isolate signaling endosomes from ES-derived MNs and analyze their molecular composition during axonal transport.

##### Development of a SILAC Protocol for ES-derived Motor Neurons

SILAC is based on the incorporation of amino acids labeled with stable isotopes into the whole proteome. In our case, three different isotopologs of arginine and lysine were used to investigate three different time points after H_C_T internalization (10, 30, and 60 min; Fig. 1*B*). Using SILAC, the experimental variability between different samples and separate MS analyses are considerably reduced compared with conventional label-free MS approaches. However, the success of SILAC relies on the complete incorporation of medium and heavy amino acids during protein turnover, and therefore, depends on the degree of cell proliferation (34). In this regard, post-mitotic cells such as neurons are potentially problematic because of the expected low percentage of incorporation during the time frame of the experiment. Nevertheless, SILAC protocols have been successfully adapted to primary neurons by adopting *ad-hoc* normalization procedures or careful optimization, such as longer culturing times to achieve acceptable percentages of incorporation (23, 26, 35).

These experimental difficulties have been bypassed by using MN-like cells, which can be indefinitely propagated in culture, and are well suited for biochemistry and high-throughput analyses. Although MN-like cells have been extensively used to study NT trafficking, MN function and pathological mechanisms (36, 37), their physiological relevance has been challenged because of major differences with primary MNs such as lack of specific neuronal markers and distinct regulation of key signaling pathways involved in neurodegeneration (24). To overcome these shortcomings, we decided to use MNs differentiated from mouse ES cells, using a modified version of the protocol described by Wichterle *et al.* (38). This well-established technique leads to the generation of 20–30% of MNs, exhibiting the same morphology, lineage markers and functional properties than primary spinal cord MNs (38–40). We obtained about 20% of MNs, as assessed by the expression of the MN specific markers HB9, Islet1/2 and choline acetyltransferase (ChAT) (supplemental Fig. S1*A* and *B*). These cells are able to internalize efficiently H_C_T, which accumulates in the perinuclear region of MNs (supplemental Fig. S1*B*) (31, 33). In contrast, H_C_T does not bind and/or undergo endocytosis in glial cells (asterisks; supplemental Fig. S1*B*). Because our aim was to analyze the composition of a relatively rare intracellular organelle, getting a high yield of MNs was essential to secure a sufficient amount of protein samples to provide reliable MS identification and quantification.

We opted for an extended incorporation kinetics spanning from ES cells to fully differentiated MNs. We started the incorporation of light (R0K0), medium (R6K4), and heavy (R10K8) amino acids into ES cells, 4 days before initiating the differentiation process (Fig. 1*B*), during which the labeled amino acids were kept in the culture media. Although at least six cell division cycles were recommended to achieve >90% of incorporation (41), we were able to achieve a percentage of incorporation close to 98% at day 2 of the differentiation process (data not shown). This high incorporation efficiency might be explained by the sustained growth of ES cells during the first 2 days of differentiation. No difference was observed in terms of ES cell morphology, cell division, or differentiation ability in the SILAC media (data not shown), confirming that the chosen SILAC conditions do not have discernable phenotypic effects on these processes (42). Notably, these labeling conditions allowed us to achieve an incorporation of nearly 100% in ES-derived MNs, with a negligible arginine to proline conversion rate, a known shortcoming of this procedure (supplemental Fig. S2). As observed for ES cells, MNs maintained in SILAC medium did not exhibit any overt morphological difference, and their ability to bind and internalize H_C_T was equivalent to control cells (data not shown).

These results indicate that we established a reliable SILAC protocol for the labeling of mouse ES cells and ES-derived MNs, which preserves the pluripotency and self-renewal properties of ES cells that retain their ability to differentiate into MNs.

##### Signaling Endosomes Purification and SILAC Quantification

To isolate signaling endosomes from ES-derived MNs, we first coupled H_C_T and GST to super-paramagnetic monocrystalline iron oxide nanoparticles (MIONs) (6, 27). To assess the specificity of our purification strategy, we performed a pilot experiment in which H_C_T-conjugated MIONs were internalized for 60 min in unlabeled ES-derived MNs. The specificity of internalization was assessed by using MIONs conjugated to glutathione S-transferase (GST), a protein that is not internalized in MNs by receptor-mediated endocytosis (data not shown). Proteins associated to GST-MIONs are potential contaminant proteins, and were labeled accordingly in supplemental Table S1. The efficiency and specificity of H_C_T-MIONs internalization in MNs was also verified by immunofluorescence (supplemental Fig. S3) and the composition of purified carriers was assessed by Western blotting. Established markers of signaling endosomes, such as cytoplasmic dynein, a microtubule-dependent molecular motor, and the small GTPase Rab5 were associated with the H_C_T-MIONs sample but not with control MIONs, showing the specificity of our purification strategy (Fig. 1*C*).

In the SILAC experiment, we followed a pulse-chase procedure, where light (L), medium (M) and heavy (H) ES-derived MNs were incubated with H_C_T-MIONs for 10 min, 30 min, and 60 min respectively (Fig. 1*B*), before being washed and mechanically lysed. Post-nuclear supernatants were mixed 1:1:1 and submitted to a magnetic isolation process, from which H_C_T-MIONs-containing endosomes were purified (6). After electrophoretic separation, protein samples underwent in-gel tryptic digestion and peptide mixtures were subjected to multiplexed MS analysis using SILAC. As multiplexing occurred prior to MS analysis, the three mixed populations of proteins from L, M, and H samples underwent identical preparation steps until they appeared as separate peptide intensity signals in the mass spectrometer. The advantage of this method is that labeling and mixing were done early in our workflow, thereby keeping technical variation to a minimum. Triplex intensity peaks in MS1 also informed on peptide amino acid composition as they revealed the number of arginine and lysine present in the peptides, a value that is used as a MS2 database search constraint by the MaxQuant/Andromeda software. MS1 signal that was not identified by MS2 database search could still be used for quantification, because MaxQuant would predict the location of peptide isotopologs in the LC-MS datafile with great accuracy through mass and retention time correlation. Thus, compared with label-free methods, fewer missing values were provided, thereby facilitating downstream analysis and interpretation of the data.

**Fig. 2.**
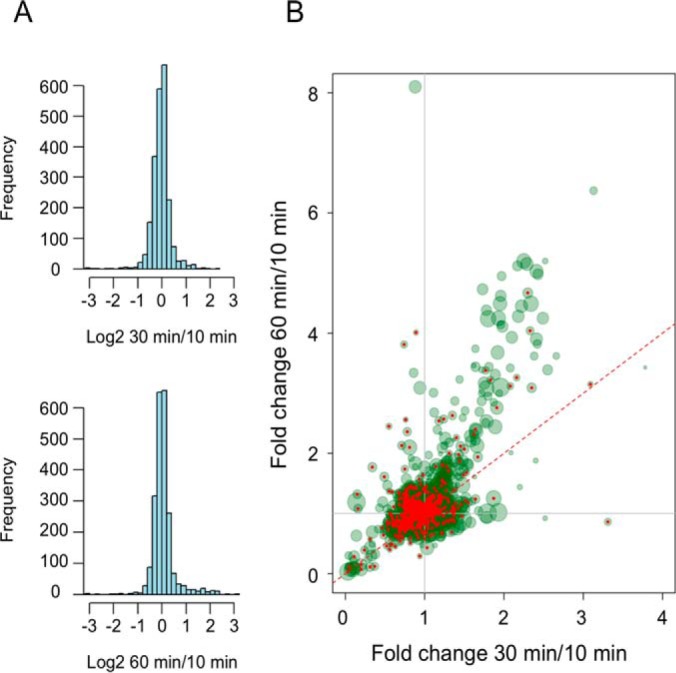
**SILAC analysis.** *A,* Normal distribution of the log2 ratios of 30 min/10 min proteome (top) and 60 min/10 min proteome (bottom). *B,* SILAC fold change (FC) scatter plot. X-axis (M/L: 30 min/10 min). Y-axis (H/L: 60 min/10 min). Proteins associated with high intensities as determined by the MaxQuant/Andromeda software are plotted with larger point sizes. Proteins on the red dotted line are characterized by identical M and H values. Potential nonspecific proteins are in red.

We identified 2306 proteins (estimated false discovery rate, FDR: 1%), of which 2262 were quantified. Three different SILAC ratios, corresponding to the three different time points of H_C_T internalization, were determined by MaxQuant: M/L, H/M, and H/L (M/L, 30 min/10 min; H/M, 60 min/30 min; H/L, 60 min/10 min). All time point log2 ratios approximately followed a normal distribution with relatively few outliers (Fig. 2*A*). Fig. 2*B* shows the distribution of the proteins as a function of their 30 min/10 min and 60 min/10 min fold changes (FC) during the time-course. In the same figure are highlighted possible contaminants (Fig. 2*B*; red dots), which were selected based on their higher abundance in the GST-MIONs sample (supplemental Table S1). These proteins might be implicated in the fluid-phase uptake of GST-MIONs and were not further analyzed in this study. The low abundance of those proteins (∼31%) compared with the total MS data underscores the specificity of our purification technique. As shown in Fig. 2*B*, the abundance ratios of most of the signaling endosome-associated proteins remained constant during internalization and transport, as exhibited by the cloud of data points close to 1.0 FC. Notably, a pool of outliers was observed on the top-right end of the plot, which corresponds to proteins that accumulate in signaling endosomes at late time points in the trafficking process. The size of each point in this plot reflects its relative intensity, with bigger point sizes relating to proteins with high intensities and therefore likely to be highly abundant in the purified signaling endosomes. In addition, proteins with high intensities were generally associated with multiple quantifications, making our data more reliable. Few potential contaminants were found in this pool of outliers, making the majority of proteins in this pool highly specific. In addition, these proteins were highly abundant and their association to the signaling endosomes was time-dependent.

##### Cell Compartment Distribution of Signaling Endosome-Containing Proteins

**Fig. 3.**
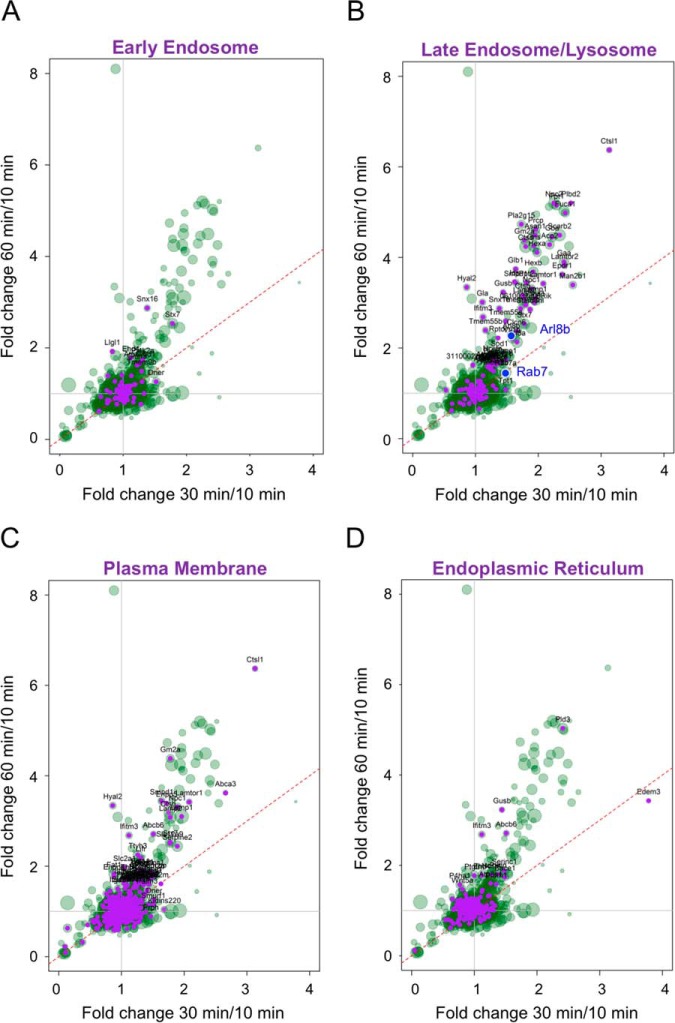
**Cell compartment distribution of signaling endosome components by Gene Ontology analysis.** QuickGO terms were overlaid onto the SILAC FC scatter plot, from which potential nonspecific proteins had been removed. *A,* Early endosome. *B,* Late endosome/Lysosome. *C,* Plasma membrane, *D,* Endoplasmic reticulum. The proteins of a given compartment are highlighted in purple and annotated with corresponding gene names. Candidates of particular interest (Rab7 and Arl8b) are highlighted in blue in *B*.

To better characterize the identified signaling endosome components, we performed gene ontology (GO) analyses on the quantified proteins. We noticed that several early endosome markers were present but most of them clustered around the cloud of data close to 1.0 FC, suggesting that they remained relatively constant throughout trafficking and signaling endosome maturation (Fig. 3*A*), as seen for the small GTPase Rab5 (60/10 ratio: 0.80; supplemental Table S1). These results confirmed previous data showing that Rab5 was associated to H_C_T-positive endosomes at early time-points after internalization, during which these organelles were stationary or displayed localized short-range movement in axons (6).

The abundance of a set of proteins clustered at the bottom left of the plot decreased with time, suggesting that they constitute a potential pool of early components enriched at 10 min of internalization (supplemental Fig. S4 and supplemental Table S2). Although these proteins might play a role in early steps of H_C_T-MIONs internalization, we focused our analyses on proteins accumulating on signaling endosomes, whose recruitment might be instrumental for their maturation and trafficking toward the cell body.

After internalization, the acquisition of the late LE marker Rab7 allows these H_C_T carriers to undergo fast axonal transport toward the cell body (Fig. 1*A*) (6). Accordingly, several LE/lysosome markers were recruited very early in the biogenesis of signaling endosomes in addition to Rab7 (Fig. 3*B*), whose abundance already increased between 10 and 30 min (30/10 ratio: 1.48; supplemental Table S1). This result fits with the observed lag phase in the onset of fast axonal transport, which usually last between 15 min and 20 min (31). SNX16, a protein of the sorting nexin family, is recruited with the early markers between 10 min and 30 min, and its abundance increases afterward (30/10, 60/30 and 60/10 ratios: 1.38, 2.06 and 2.87, respectively; supplemental Table S1), which is in accordance with its distribution in both early and late endosomes and its role in the regulation of trafficking between these two compartments (43). Therefore, the choice of a narrow time course (10, 30 and 60 min) allowed us to provide more accurate information about the kinetics of signaling endosome transition from an early to a late stage. The observed acquisition of LE determinants was confirmed by our enrichment analysis showing that LE/lysosome markers accumulated in axonal retrograde carriers. Indeed, using a hypergeometric test, we showed that among specific signaling endosome proteins, 137 of them were part of LE/lysosome, resulting in a highly significant enrichment (*p* value, 7.853 × 10^−49^).

Overall, we found that signaling endosomes contained several plasma membrane proteins showing a significant time-dependent ratio change (Fig. 3*C*). Most of these proteins were also found in LE/lysosome compartments, reflecting the continuous cycling of many plasma membrane proteins through LE. In addition, GO analysis indicated that numerous endoplasmic reticulum (ER) proteins were present in signaling endosomes (Fig. 3*D*), which could be because of the extensive contact sites existing between ER and endosomes (44) and plasma membrane and ER (45). Several proteins enlisted in the ER and plasma membrane groups were also found in our list of potential contaminants, together with proteins normally localized in the nucleus and mitochondria (supplemental Table S1). Their presence among specific carrier components could be explained at least in part by the extensive contact sites existing between different organelles (45), but also by the multiple compartment localization of some of these proteins, such as superoxide dismutase 1 (SOD1), whose mutations lead to ALS (25) (supplemental Table S1). However, very few specific proteins from this outlier population belong to mitochondrial or nuclear compartments (supplemental Fig. S5*A* and S5*B*, respectively), further confirming their specificity.

Several ribosomal proteins were identified in purified signaling endosomes (supplemental Table S1). Although their high abundance makes them often come up as false positive in MS experiments, ribosomal proteins might also be genuine components of retrograde carriers, as suggested by the recent report showing the association of ribosomes with motile endosomes, which links mRNA translation to endosome transport (46–48).

Altogether, our data suggest that after H_C_T-MIONs internalization, signaling endosomes were rapidly enriched in LE/lysosomal markers, which show their maturation during their journey to the soma.

##### Enrichment of Signaling Endosomes with Protein Implicated in Human Disease

Several studies have shown that neurodegeneration may be linked to axonal transport deficits caused by mutations in genes coding for proteins involved in organelle trafficking, such as molecular motors, motor adaptors and regulators, and cytoskeletal components (7, 8, 49). To investigate whether signaling endosomes are significantly enriched for proteins associated with human pathologies, we performed enrichment analysis using GeneGO and Metacore ontology for diseases. As shown in Table I, signaling endosomes were significantly enriched (*p* value <0.05) in proteins known to be involved in neurological disorders, such as Alzheimer's disease, Huntington's disease, motor neuron disease including ALS and tauopathies, which have all been associated with altered axonal transport. This result confirmed that several signaling endosome proteins are linked to neurodegeneration, and that an alteration of their temporal-dependent association with the carriers might lead to axonal transport deficits.

**Table I:** Association of signaling endosome components with human pathologies. Disease enrichment analysis was performed with Metacore using a hypergeometric test. The ratios between endosomal protein-coding genes and independent genes indicate the possible association of any given gene with neurological diseases. Selected genesets are listed in order of their statistical significance after Benjamini-Hochberg correction. p values < 0.05 are significant. Neurological diseases associated with axonal transport deficits are highlighted in bold. Neurological infections are marked with asterisks. Adj.p.value: adjusted p value

Disease name	adj.p.val	Ratio
**Central nervous system Diseases**	1.89E-47	459/3069
**Brain diseases**	6.73E-46	435/2880
Schizophrenia	6.25E-43	203/914
Schizophrenia and disorders with psychotic features	9.56E-43	203/918
**Neurodegenerative diseases**	2.74E-39	336/2104
Mental disorders	3.50E-39	281/1614
**Nervous system diseases**	3.50E-39	665/5522
Psychiatry and psychology	8.79E-36	307/1919
**Dementia**	9.48E-32	255/1536
**Tauopathies**	6.13E-29	206/1166
**Alzheimer's disease**	2.04E-27	201/1153
Virus diseases*	1.68E-17	215/1531
**TDP-43 proteinopathies**	2.55E-17	54/180
**Amyotrophic lateral sclerosis**	9.00E-17	53/179
Heredodegenerative disorders, nervous system	1.19E-16	163/1064
Glioblastoma	7.15E-16	311/2579
Astrocytoma	9.92E-16	315/2628
Epilepsy	1.09E-15	90/455
Basal ganglia diseases	5.70E-15	129/799
Movement disorders	3.01E-14	135/871
**Neuromuscular diseases**	1.31E-13	176/1278
**Spinal cord diseases**	2.14E-13	72/352
Neurologic manifestations	2.38E-13	204/1566
**Motor neuron disease**	2.76E-13	59/257
Tay-Sachs disease	8.24E-13	12/12
Dyskinesias	8.98E-13	117/745
Brain diseases, metabolic	1.71E-12	62/290
Bacterial infections and mycoses*	1.76E-12	175/1307
Glioma	2.44E-12	534/5313
RNA virus infections*	4.27E-12	148/1055
Brain diseases, metabolic, inborn	9.98E-12	59/279
Neoplasms, neuroepithelial	1.00E-11	554/5596
Infection*	7.58E-11	157/1185
**Down syndrome**	1.94E-10	42/173
**Huntington's disease**	4.97E-09	75/461
Lysosomal storage diseases, Nervous system	5.14E-09	23/67
**Parkinsonian disorders**	2.45E-05	58/405
**Frontotemporal lobar Degeneration**	2.24E-04	18/80
**Frontotemporal dementia**	2.24E-04	18/80

**Fig. 4.**
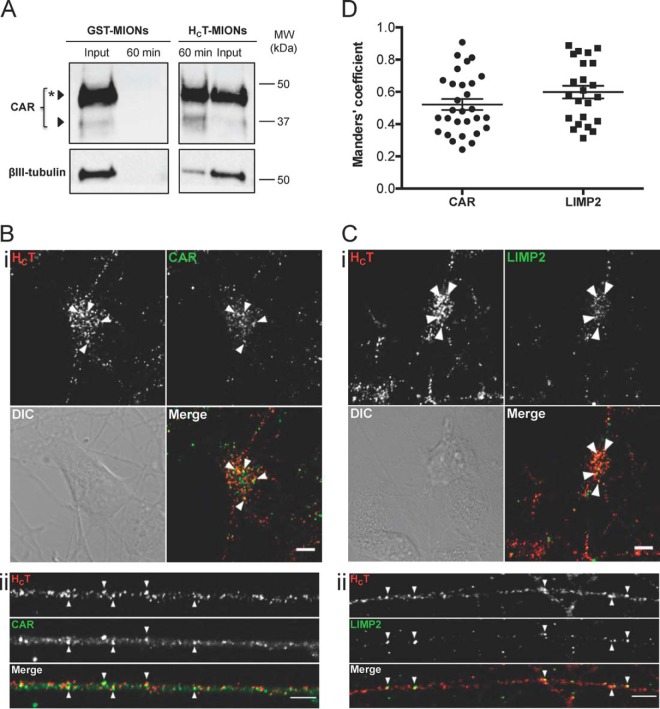
**Association of viral receptors with signaling endosomes.** *A*, DIV3 ES-derived MNs were incubated for 60 min at 37 °C with H_C_T- or GST-conjugated MIONs, acid-washed and submitted to magnetic endosome purification. The inputs (post-nuclear supernatants, 2% of total) and the whole 60 min eluates were then separated by SDS-PAGE and analyzed by western blotting to assess the presence of CAR (top). βIII-tubulin (bottom) was used as a loading control. A star indicates the glycosylated form of CAR, a modification that could strengthen the binding of CAV and stabilize the complex in the carriers for transport. MW: molecular weight. *B*, *C*, DIV3 ES-derived MNs were incubated with H_C_T for 60 min at 37 °C, acid-washed, fixed and processed by immunofluorescence to detect HA-H_C_T-MIONs and CAR (*B*) or LIMP2 (*C*). Single plane images of cell bodies (i) and neurites (ii) are shown. Examples of organelles positive for H_C_T and CAR or LIMP2 are indicated by arrowheads. Significant co-distribution was noticed in somas and neurites for both candidates. Scale bars: 5 μm. The overall degree of colocalization between H_C_T and CAR or LIMP2 was quantified and the corresponding Menders coefficients are shown in *D*. Error bar: standard error of the mean.

Bacterial and viral entry into the central nervous system (CNS) is also likely to be associated with signaling endosome trafficking (4), because several receptors for neurotropic pathogens are found in these organelles (Table II). Indeed, many pathogens have the ability to hijack the axonal transport pathway to access the CNS, tetanus neurotoxin being one of the best examples (50). We found that the synaptic vesicle proteins synaptotagmins 1 and 2 and SV2A, which function as the receptors of botulinum neurotoxins, were associated to signaling endosomes, a finding which may explain the central effects of these neurotoxins (51). Similarly, it had been shown that several neurotropic viruses bind their receptors at axon terminals and enter endosomes traveling to the neuron soma (4). Our study shows that signaling endosomes host proteins involved in viral CNS infections (Table II). Indeed, the coxsackie and adenovirus (CAV) receptor CAR is a highly abundant signaling endosome component (Fig. 4*A*, (52)) and like the enterovirus 71 receptor LIMP2 (lysosomal integral membrane protein 2) (53), was found to colocalize with H_C_T in axonal carriers (Fig. 4*A* and 4*B*), as shown in the quantification displayed in Fig. 4*D.* Importantly, these results are in accordance with previous studies showing that CAV, enterovirus 71 and poliovirus undergo axonal retrograde transport in MNs (4, 52, 54).

**Table II:** Signaling endosomes and infection. Neurotropic pathogen receptors present in signaling endosomes are listed together with their corresponding viral or bacterial pathogen. Gene names are indicated in brackets. With the exception of LIMP2, the abundance of these receptors in signaling endosomes remains constant during axonal transport

Pathogenic agent	Receptor *(gene)*
Adenovirus	CAR *(Cxadr)*
Coxsackievirus B	CAR *(Cxadr)*
Poliovirus	PVR *(PVR)*
Herpes Simplex virus	Nectin-1 *(Pvrl1)*
Pseudorabies virus	Nectin-1 *(Pvrl1)*
Rabies virus	p75NTR *(Ngfr)*
Rabies virus	NCAM *(Ncam)*
Enterovirus 71	LIMP2 *(Scarb2)*
Chikungunya virus	Prohibitin (*Phb*)
Hendra virus/Nipah virus	EphrinB2 (*Ephb2*)
Nipah virus	EphrinB3 (*Ephb3*)
Junìn virus	CACNA2D2 *(Cacna2d2)*
Botulinum neurotoxin B/G/DC	Synaptotagmin 1/2 *(Syt1/2)*
Botulinum neurotoxin A/E/F	SV2A *(Sv2a)*

##### Dynamic Changes of Signaling Endosomes Proteins During Axonal Transport

After assessing the molecular composition of signaling endosomes, we investigated the dynamic changes occurring in their associated proteins during axonal transport. For this analysis, we focused on proteins that showed a temporal dependence, chosen according to the significance score (significance B) of their fold change ratio. Significance B was calculated following a MaxQuant method based on signal intensities, with the more significant fold changes corresponding to the more abundant proteins (55). These calculations allowed us to consider the group of proteins that had significant increased and/or decreased ratios at 60 or 30 min compared with the 10 min time point (supplemental Fig. S6*A* and S6*B*; supplemental Table S1). We found that 260 proteins (plus two isoforms) met these criteria (supplemental Fig. S6*C* and supplemental Table S3). This dataset included most of the proteins that would constitute candidates of interest in the maturation of signaling endosomes as indicated by their gene annotations (Fig. 3*B* and supplemental Table S3). Enrichment analysis revealed that several proteins are involved in intracellular transport, lysosome organization, neuronal development and differentiation (Table III). The presence of the latter group of proteins is not surprising because axon guidance and neurite outgrowth rely on axonal transport (56). Overall, changing proteins could be grouped into six clusters, following their trend change during the time-course, *i.e.* exhibiting a constant increase or decrease throughout the kinetics, exhibiting increasing then decreasing abundance and vice-versa, or exhibiting temporal dependence at only one time point, as displayed by the heatmap in supplemental Fig. S7.

**Table III:** Dynamic changes of signaling endosome components and biological processes. Biological processes enrichment was performed similarly to the disease enrichment analysis shown in Table II using a hypergeometric test and the Metacore software. Biological processes that involve transport mechanisms are highlighted in bold. GO: gene ontology. Adj.p.value: adjusted p value

GO Processes name	ad.p.val	Ratio
**Cellular component organization**	3.24E-17	158/6165
Small molecule metabolic process	1.33E-14	92/2752
**Transport**	1.52E-12	123/4781
**Nervous system development**	1.66E-12	91/2979
**Cell projection organization**	2.35E-11	55/1361
**Cell morphogenesis involved in neuron differentiation**	2.61E-11	39/727
**Neuron development**	2.89E-11	51/1201
**Neuron projection development**	3.82E-11	45/966
**Neuron projection morphogenesis**	5.51E-11	39/750
**Cellular localization**	5.51E-11	83/2766
Catabolic process	4.63E-10	69/2162
**Axon development**	4.85E-10	37/738
**Brain development**	5.22E-10	44/1016
**Neuron differentiation**	2.13E-09	53/1462
**Generation of neurons**	4.50E-09	63/1979
Sphingolipid catabolic process	5.43E-09	9/28
**Axonogenesis**	7.38E-09	33/664
**Vesicle-mediated transport**	9.91E-09	53/1533
Membrane lipid catabolic process	9.91E-09	9/30
**Neurogenesis**	1.09E-08	64/2081
**Central nervous system development**	1.74E-08	48/1326
**Macromolecule localization**	2.49E-08	72/2549
**Secretion by cell**	3.07E-08	32/669
**Establishment of localization in cell**	3.10E-08	67/2297
**Regulation of neuron projection development**	4.81E-08	27/497
**Exocytosis**	1.21E-07	24/415
Cellular catabolic process	1.44E-07	55/1766
**Lysosome organization**	2.35E-07	10/58
**Lytic vacuole organization**	2.35E-07	10/58
**Neuron projection guidance**	2.39E-07	27/541
**Axon guidance**	2.39E-07	27/541
**Membrane organization**	4.73E-07	41/1153

We focused on LE/lysosome proteins (Fig. 3*B*) whose abundance significantly increased from 10 to 30 min and/or from 10 to 60 min. In fact, the recruitment of many LE components continued to increase after 30 min (Fig. *3**B* and supplemental Table S3). Among these, we identified well-known endolysosomal proteins, such as members of the lysosome-associated membrane glycoprotein (LAMP) and LIMP families (LAMP1, LAMP2: 30/10 ratios: 1.96 and 1.78; 60/10 ratios: 3.10 and 3.08; 60/30 ratios: 1.59 and 1.73 respectively; and LIMP2: 30/10 ratio: 2.34; 60/10 ratio: 4.49; 60/30 ratio: 1.94, supplemental Table S3). Vti1b, a v-SNARE involved in LE homotypic fusion and in LE-lysosome fusion (57), was also found in signaling endosomes, and exhibited progressive enrichment in these organelles (30/10 ratio: 1.58; 60/10 ratio: 2.20; supplemental Table S3). In addition, we identified the small GTPase Arl8b, which exhibited similar ratios (30/10 ratio: 1.57; 60/10 ratio: 2.29; 60/30 ratio: 1.47, supplemental Table S3), consistent with its key role in lysosome biogenesis and motility and LE/lysosome fusion (30, 58, 59). Arl8b is significantly recruited to signaling endosomes during maturation, which may be related to its putative function in axonal transport (60). Among the increasing late components, we also identified the LE/lysosome adaptor proteins LAMTOR1 and 2 (30/10 ratios: 2.08 and 2.42; 60/10 ratios: 3.42 and 3.82, respectively; supplemental Table S3), which were implicated in signal transduction from this compartment (61). The LE marker Rab7 displayed a significant increase only at 30 min and remained relatively stable thereafter (30/10 ratio: 1.48; 60/10 ratio: 1.47; supplemental Table S3), which suggests that it is a stable component of retrograde carriers upon its recruitment at axon terminals (Fig. 1*A*).

##### Biological Validation of Identified Signaling Endosome Components and Their Recruitment Kinetics

**Fig. 5.**
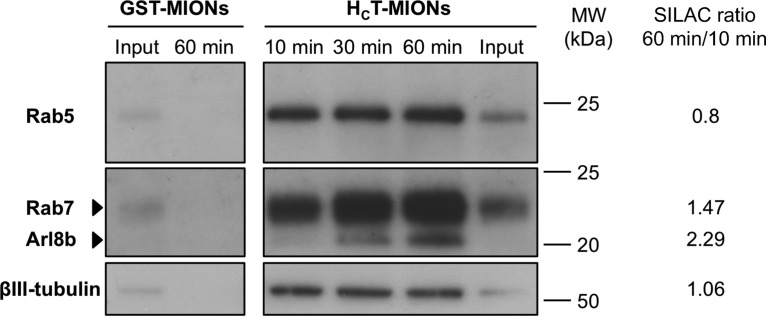
**Candidate validation by western blotting.** DIV3 ES-derived MNs were incubated for 10 min at 37 °C with H_C_T- or GST-conjugated MIONs, then chased for 10, 30, or 60 min at 37 °C and purified by magnetic separation. Input (post-nuclear supernatant, 1% of total) and 10/30/60 min H_C_T-MIONs and GST-MIONs eluates (33% of total) were then separated by 12% Bis-Tris SDS-PAGE to improve the separation of closely related low molecular weight proteins, and analyzed by Western blotting to assess the presence of Rab5, Rab7, and Arl8b (top). βIII-tubulin was used as a loading control. The 60 min/10 min SILAC ratios were added for temporal-change comparison.

To confirm the SILAC data, we selected specific signaling endosome components and checked for their presence and temporal-dependent changes by western blotting and immunofluorescence. To independently confirm the SILAC results, we repeated the internalization kinetics and purification experiment in label-free conditions. ES-derived MNs were pulse-chased with H_C_T-MIONs for 10, 30 and 60 min, or GST-MIONs for 60 min. We confirmed that the early endosome marker Rab5 was specifically enriched in samples isolated with H_C_T-MIONs, but not in GST-MION controls (Fig. 5), further emphasizing the specificity of our purification strategy. Notably, the association of Rab5 to H_C_T-MION-containing organelles did not change with time (Fig. 5). In agreement with its essential role in the onset of axonal retrograde transport, we observed a time-dependent recruitment of Rab7, with a strong increase especially between 10 and 30 min (Fig. 5), a change that correlates well with the observed 60/10 fold-change of 1.47 obtained with the SILAC experiment (supplemental Table S3). The LE/lysosomal protein Arl8b was also progressively recruited to the H_C_T-retrograde carriers, as showed by a 60/10 SILAC ratio of 2.29 (Fig. 5 and supplemental Table S3). The abundance of these proteins was normalized to βIII-tubulin, a neuronal-specific marker, which remains constant during transport (60/10 ratio: 1.06; Fig. 5 and supplemental Table S1). Western blot analysis of the same samples confirmed the time-dependent recruitment of the LE v-SNARE Vti1b to signaling endosomes from 10 min post-internalization (data not shown), a result that confirms the increase of SILAC ratios during endosomal maturation (60/10 ratio: 2.20, supplemental Table S3).

**Fig. 6.**
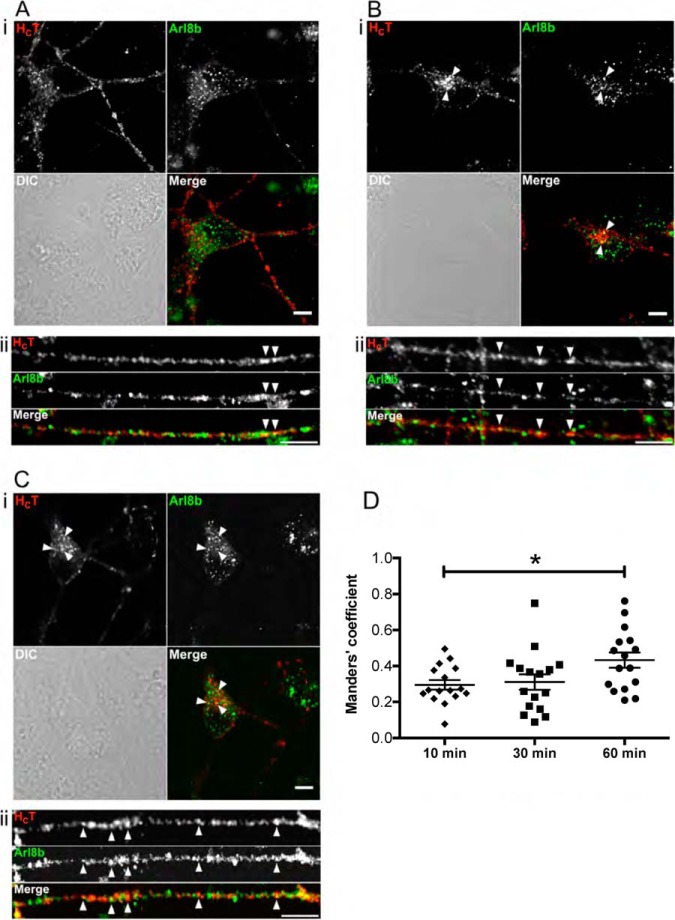
**Validation of Arl8b localization on signaling endosomes by immunofluorescence.** DIV3 ES-derived MNs were incubated for 10 min at 37 °C with H_C_T, and then chased for 10 (*A*), 30 (*B*), or 60 min (*C*) at 37 °C, acid-washed, fixed and processed by immunofluorescence to detect HA-H_C_T and Arl8b. Single plane images of cell bodies (i) and neurites (ii) are shown. Example of organelles positive for H_C_T and Arl8b are indicated by arrowheads. Codistribution was noticed in neurites at 10 min chase. The degree of H_C_T-Arl8b colocalization increased both in neurites and the cell body over time, especially in the perinuclear H_C_T accumulation sites. Scale bars: 5 μm. The degree of colocalization between H_C_T and Arl8b increases significantly (*) at 60 min chase, as shown by the Menders coefficients in D. * < 0.05. Error bar: standard error of the mean.

Furthermore, the SILAC results were independently confirmed by visualizing the recruitment of selected MS hits to H_C_T-containing organelles using immunofluorescence. As shown in Fig. 6, Arl8b did not display overt colocalization with H_C_T in the soma at 10 min chase, whereas co-distribution was observed in neurites (Figs. 6*A*, i and ii). However, the degree of H_C_T-colocalization with Arl8b increased over time both in neurites and the cell body (Fig. 6*B*, i and ii), and rose significantly at 60 min, especially in the perinuclear region, where H_C_T accumulated (Figs. 6*C,* i and ii; Fig. 6*D*).

Taken together, these experiments show that our purification strategy and SILAC approach are highly specific. We confirmed that the late marker Arl8b is progressively recruited to signaling endosomes in motor neurons. Our observations suggest that this novel component might play a pivotal role in axonal retrograde transport and/or sorting events taking place in the soma, with important consequences for the downstream signaling and fate of the cargoes of signaling endosomes.

## DISCUSSION

In this report, we describe the first quantitative proteomic analysis of signaling endosomes isolated from MNs. Our results provide novel insights on the molecular composition of these organelles and the spatiotemporal dynamics of axonal transport. Our SILAC data show that signaling endosomes acquire LE markers during their transport to the soma, and provide new information about their maturation mechanism. Signaling endosomes acquire LE markers between 10 and 30 min, a kinetics that closely overlap with the onset of fast axonal transport, which occurs 20 min post-internalization (6, 62).

Signaling endosome maturation was confirmed by the increasing recruitment of several LE/lysosome proteins, such as Arl8b and Vti1b (Figs. 3*B* and 6; supplemental Table S1). A possible explanation of the continuous increase of these proteins during the time course of our analysis is that their recruitment is functionally linked to the initiation and/or steady-state support of fast retrograde transport, as previously reported for Rab7 (6). Alternatively, the accumulation of H_C_T-MIONs in Vti1b- (data not shown) and Arl8b-positive organelles (Figs. 5 and 6) might indicate the acquisition of fusion competence in preparation for sorting and/or fusion events occurring upon their arrival in the MN soma. Although Arl8b and Vti1b play essential roles in LE/lysosome fusion (57), the transport and somatic sorting compartments entered by H_C_T are not classical LE/lysosomes, because H_C_T carriers are not acidified during transport (33). Because full length TeNT is able to translocate through the endosomal membrane into the cytoplasm at acidic pH (50), blocking the acidification of these organelles traps TeNT in the endosomal lumen, halting its transfer to adjacent neurons (31, 33).

Several distinct functions have been described for Arl8b, including lysosome transport and fusion (30, 58, 59, 63), and homotypic fusion and vacuole protein sorting (HOPS) complex recruitment (63). Our results suggest that Arl8b may play a role in the somatic sorting of signaling endosome cargo, such as NT receptor complexes and H_C_T. Previous data showing a possible role of Arl8b in axonal transport (60) further strengthens our hypothesis. Because this route controls the down-regulation of ligand-activated receptor complexes and their signaling (28), the elucidation of the role of Arl8b in this pathway might unveil key mechanisms involved in the regulation of signaling endosome dynamics.

Our findings could provide novel insights on the underlying mechanisms linking LE/lysosomal deficits to neurodegeneration, as many proteins associated with LE/lysosome trafficking and/or function are altered in neurological diseases (49). Importantly, we showed that purified signaling endosomes were enriched in proteins involved in neurological conditions in which axonal transport deficits have been found, such as ALS, Alzheimer's, Huntington's and Parkinson's diseases, to name a few (Table I). In Parkinson's disease, mutations of LE/lysosome proteins and motor complexes, such as LRRK2, Vps35, DCTN1, have been previously documented (8, 49), suggesting that LE sorting defect and axonal transport disruption could play a causal role in this pathology. Strikingly, the lysosomal protein LIMP2 displayed a temporal-dependent incorporation into signaling endosomes (Fig. 3*B*, 4*C* and supplemental Table S1). This finding might have a wider physiological importance, because genetic variations in the LIMP2 coding gene (*Scarb2*) have been linked to the development of synucleinopathies (64). Given the variety of CNS pathologies highlighted by our enrichment analysis, additional signaling endosome components might be linked to other neurological diseases. In this regard, it would be crucial to investigate putative differences in the molecular composition and dynamics of signaling endosomes in different neurological pathologies. Our SILAC labeling strategy could easily be applied to the wide array of ES cells isolated from different mouse models of neurodegeneration currently available. Mutant ES cells could be used to generate differentiated neurons in SILAC media, from which axonal signaling endosomes could be isolated. The comparative analysis of the proteome of these organelles derived from wild type and mutant neurons could be invaluable to assess quantitative and temporal changes occurring in disease conditions. Complementary to this approach, human induced pluripotent cell-derived MNs (hiPSC MNs) (65) may open the way for further applications of our SILAC protocols to study molecular mechanisms leading to ALS and neurodegeneration in human cells.

Our proteomic analyses revealed that signaling endosomes carry many virus and virulence factor receptors (Table II), supporting the hypothesis that many neurotropic viruses hijack the signaling endosome pathway to enter the CNS (4). We confirmed the presence of the coxsackie and adenovirus receptor CAR in signaling endosomes and we showed that the majority of this receptor is glycosylated (Fig. 4*A*), a modification that could stabilize virus binding (66) and therefore ensure its efficient transport to the soma.

Along with CAR and poliovirus receptors, in our analysis we found nectin-1, the receptor of herpes simplex viruses 1 and 2, two viruses that are also known to undergo retrograde transport mainly in sensory neurons (67). The pseudorabies virus receptor was also present in our dataset, in accordance with its retrograde transport in neurons (67). Notably, two of the three described receptors for rabies virus were also present in signaling endosomes, p75^NTR^ and neural cell adhesion molecule (NCAM), a result consistent with the observed axonal retrograde transport of rabies virus in MNs (68). Furthermore, the presence of LIMP2 in the carriers suggests that enterovirus 71, which has been shown to bind this protein on the cell surface (54), might also enter and spread through the CNS in signaling endosomes (69). Emergent viruses such as Chikungunya, Hendra or Nipah are likely to use the same entry pathway because their receptors are also present in this compartment (Table II). The list of receptors we provided here is non exhaustive and may be expanded as novel receptors of CNS targeted viruses will be characterized. In this regard, we confirmed the presence of nidogen 1, a basal membrane protein, which has recently been shown to act as a receptor for TeNT (70), in our signaling endosome analysis (data not shown).

In conclusion, in this report we provide an optimized SILAC labeling strategy for ES-derived MNs and a reliable method for the purification and proteomic mapping of signaling endosomes. Our data constitute a valuable resource to further our understanding of the molecular composition and dynamics of these organelles and provide new insights on the mechanism regulating their progression along the axonal transport route, with direct links to a wide range of neurological pathologies, including CNS infections and neurodegenerative diseases.

## Supplementary Material

Supplemental Data

## Footnotes

^1^ The abbreviations used are:

MN: motor neuron
ALS: amyotrophic lateral sclerosis
BSA: bovine serum albumin
CNS: central nervous system
DIV: days *in vitro*
EB: embryoid bodies
ER: endoplasmic reticulum
ES: embryonic stem
FC: fold change
GO: gene ontology
GST: glutathione-S-transferase
H: heavy
HA: haemagglutinin
HBSS: Hank's balanced buffered salt solution
H_C_T: the binding fragment of tetanus neurotoxin
KOSR: KnockOut™ serum replacement
L: light
LE: late endosome
MIONs: monocrystalline iron oxide nanoparticles
NT: neurotrophin
RA: retinoic acid
SILAC: stable isotope labeling with amino acids in cell culture
SNARE: soluble NSF attachment protein receptor.
