# Supplementary material for: Analysis of Signaling Endosome Composition and Dynamics Using SILAC in Embryonic Stem Cell-Derived Neurons
Source: Mol Cell Proteomics. 2016 Feb;15(2):542–57. doi: 10.1074/mcp.M115.051649 (PMC4739672; doi:10.1074/mcp.M115.051649)
Supplement: Supplemental Data [file supp_15_2_542__index.html]

Analysis of signaling endosome composition and dynamics using SILAC in embryonic stem cell-derived neurons — Analysis of Signaling Endosome Composition and Dynamics Using SILAC in Embryonic Stem Cell-Derived Neurons — Spatio-Temporal Characterization of Signaling Endosomes — Supplemental Data 

# Analysis of Signaling Endosome Composition and Dynamics Using SILAC in Embryonic Stem Cell-Derived Neurons

## Supplemental Data

- Supplemental Figures (.pdf, 15.1 MB) - Supplemental Figures
- Supplemental Material (.pdf, 144 KB) - Supplemental Material: supplemental figure and table legends
- Table S1 (.xlsx, 483 KB) - Supplemental Table S1
- Table S2 (.xlsx, 42 KB) - Supplemental Table S2
- Table S3 (.xlsx, 118 KB) - Supplemental Table S3
