# Supplementary material for: Analysis of Signaling Endosome Composition and Dynamics Using SILAC in Embryonic Stem Cell-Derived Neurons
Source: Mol Cell Proteomics. 2016 Feb;15(2):542–57. doi: 10.1074/mcp.M115.051649 (PMC4739672; doi:10.1074/mcp.M115.051649)

Figure S1 Debaisieux et al.

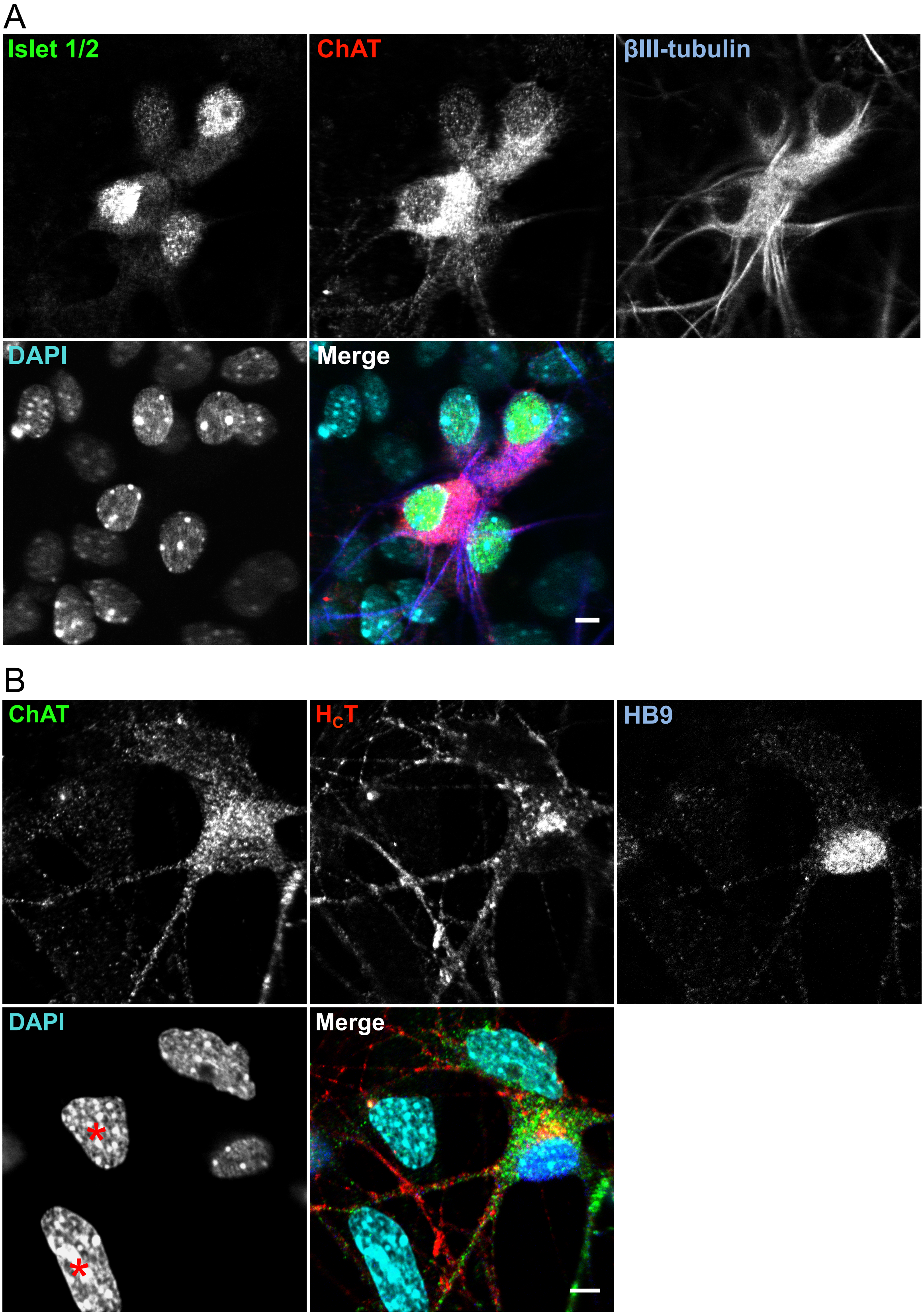

Figure S2 Debaisieux et al.

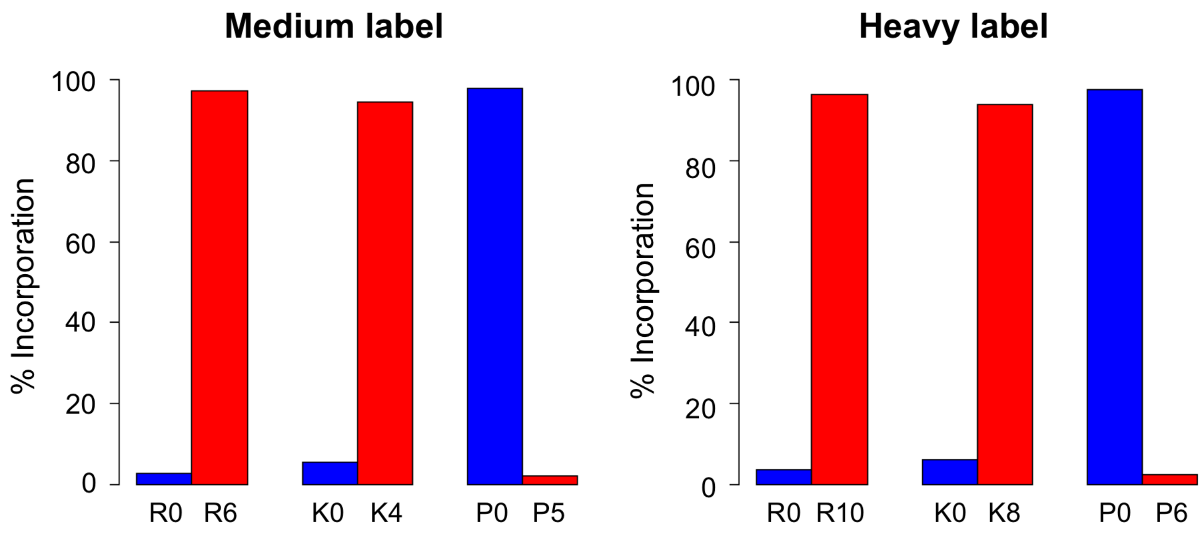

Figure S3 Debaisieux et al.

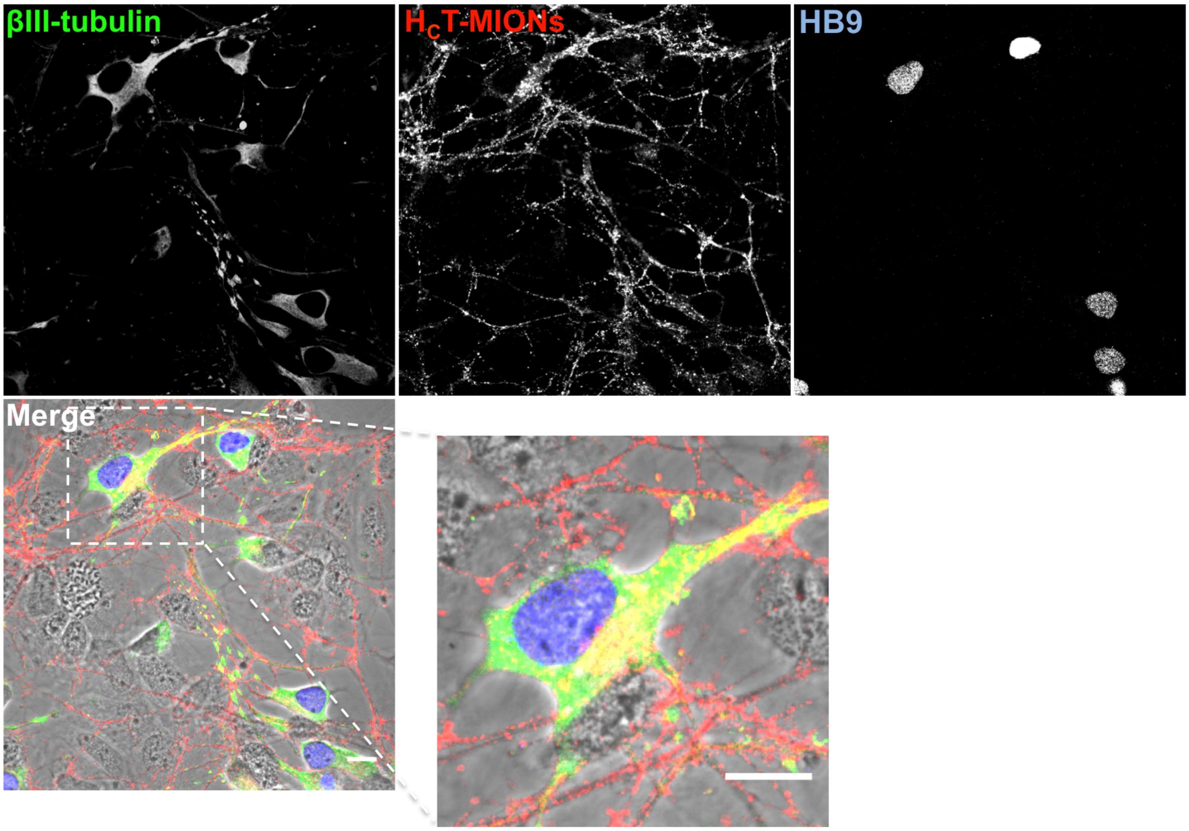

Figure S4 Debaisieux et al.

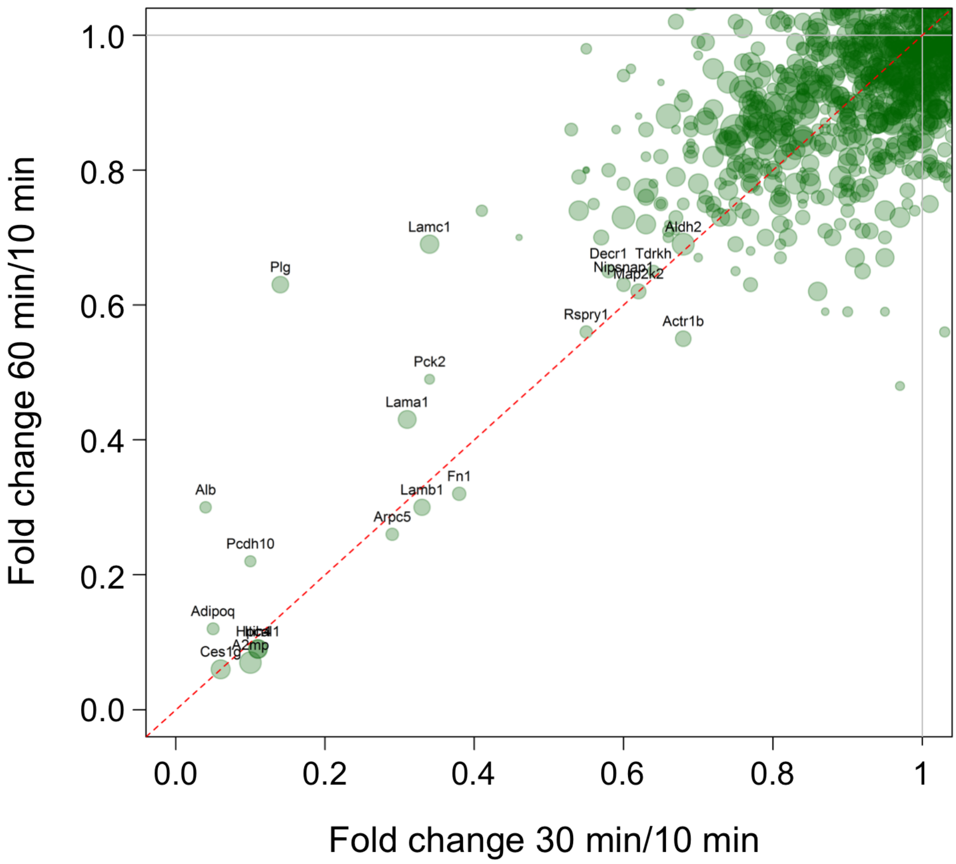

Figure S5 Debaisieux et al.

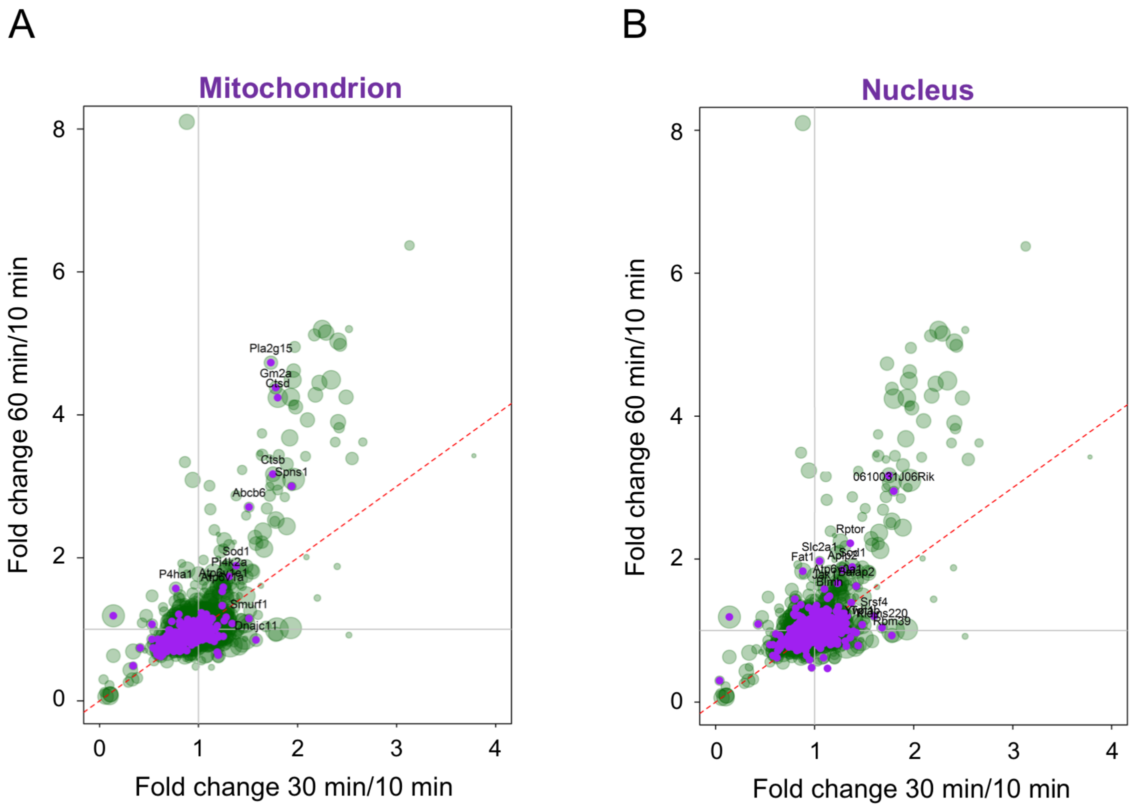

Figure S6 Debaisieux et al.

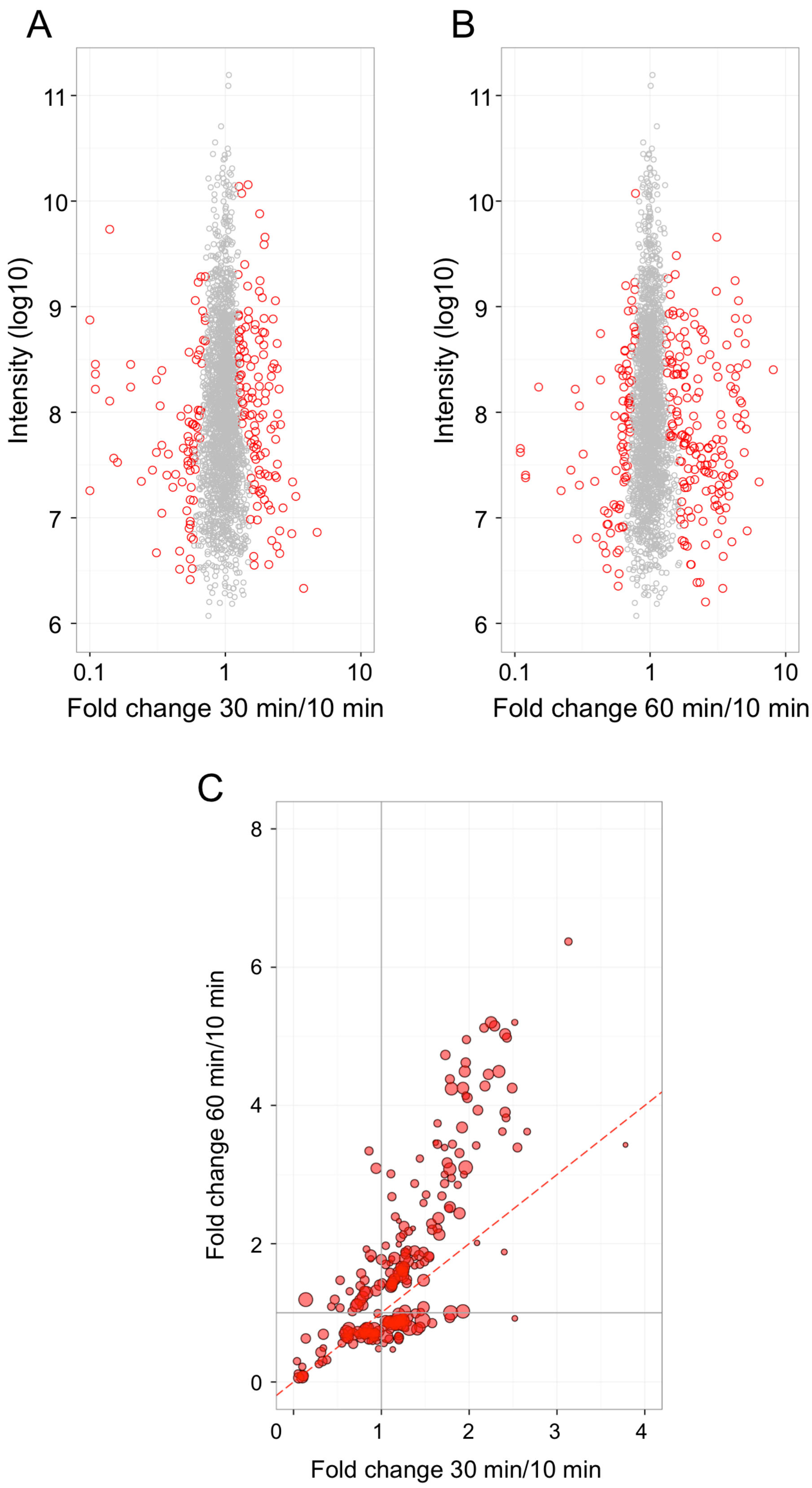

Figure S7 Debaisieux et al.

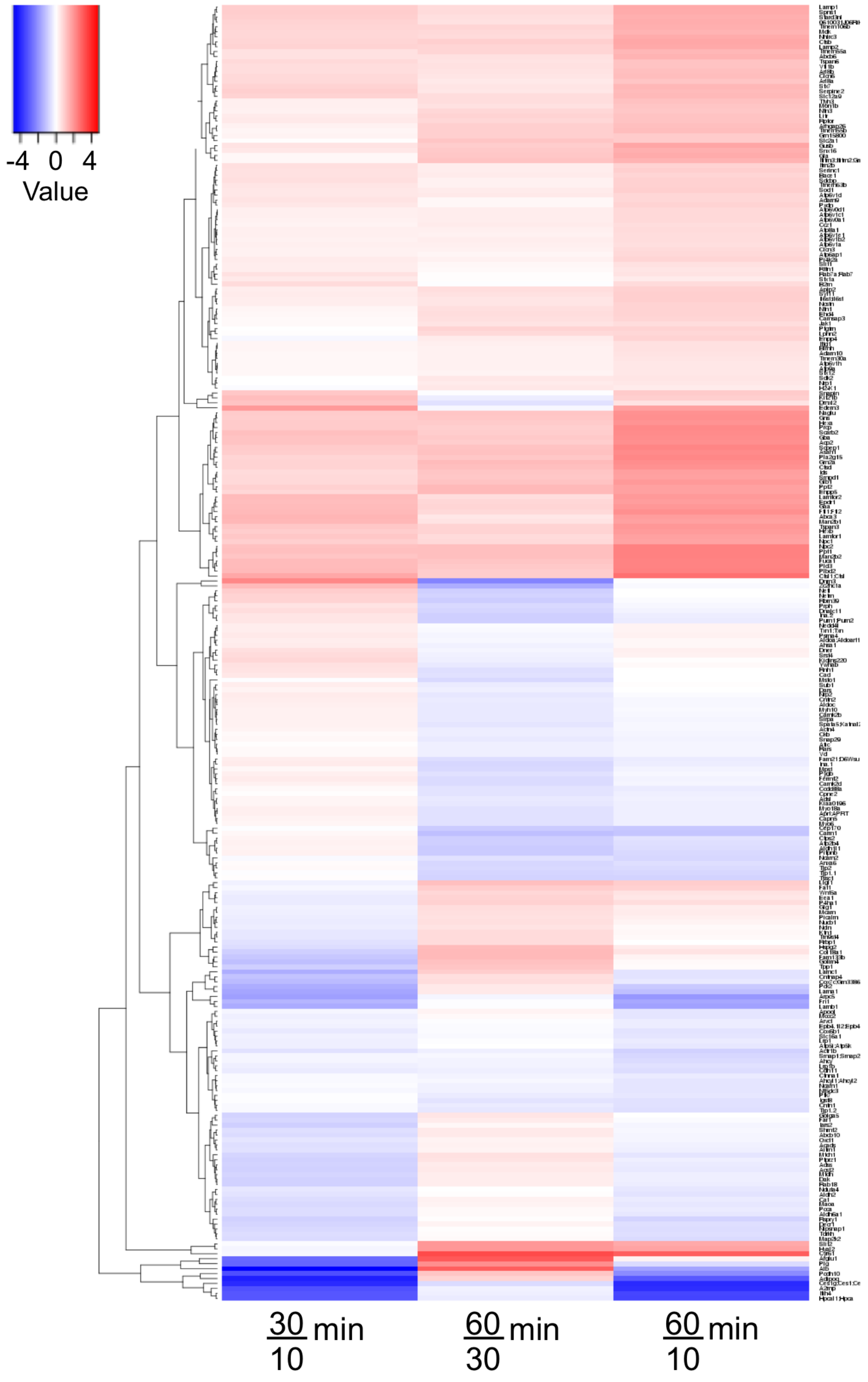

Supplement: Supplemental Data [file 10.1074_M115.051649_mcp.M115.051649-1.pdf]
