# Supplementary material for: Analysis of Signaling Endosome Composition and Dynamics Using SILAC in Embryonic Stem Cell-Derived Neurons
Source: Mol Cell Proteomics. 2016 Feb;15(2):542–57. doi: 10.1074/mcp.M115.051649 (PMC4739672; doi:10.1074/mcp.M115.051649)
Supplement: Supplemental Data [file 10.1074_M115.051649_mcp.M115.051649-2.pdf]

Supplemental Experimental Procedures

Quantification of incorporation – The percentage of incorporation of the medium (R6K4) and heavy (R10K8) amino acids was assessed at two separate time-points during ES culture and differentiation (ES proliferation day 4 and ES differentiation day 2) (data no shown) and in differentiated MNs. MNs were collected, lysed, proteins were separated on SDS-PAGE and analyzed by LC-MS/MS as described in the main text. The data was searched using MaxQuant/Andromeda, but an additional label for H/M L-proline was established to assess the percentage of conversion of H/M L-arginine to H/M L-proline. MaxQuant derived intensities for peptides containing R6K4 or R10K8 were added and compared to the combined intensities of R0K0 peptides to determine the percentage of incorporation. The same strategy was used to calculate the level of L-arginine->L-proline conversion (see supplemental Fig. *S*2).

Assessment of H_CT or H_CT-MIONs internalization by confocal microscopy - To assess the efficiency of H_CT or H_CT-MIONs internalization in differentiated MNs, DIV3 ES-derived MNs were incubated with HA-tagged H_CT or HA-tagged H_CT-MIONs for 60 min at 37 °C in complete MN medium. After internalization, MNs were cooled on ice, acid-washed and then further washed with HBSS pH 7.4. Cells were fixed in 4% paraformaldehyde in HBSS and processed for immunofluorescence, as described in the main text. Coverslips were incubated overnight at 4 °C with the following primary antibodies diluted in blocking buffer: rat anti-HA (Roche, 1:1,000), rabbit anti-HB9 (Abcam, 1:10,000), mouse anti-Islet1/2 (DSHB, 1:200), goat anti-ChAT (Chemicon, 1:100) and mouse anti-βIII tubulin (Covance, 1:1,000). Samples were then washed and incubated with AlexaFluor-conjugated secondary antibodies (Life Technologies, 1:500) for 1 h at 25 °C, followed by 4',6-diamidino-2-phenylindol**e** **(**DAPI) staining (Sigma-Aldrich, 1:5,000). After several washes, coverslips were mounted in mowiol. Confocal images were acquired using a Zeiss LSM780 equipped with a Plan-Apochromat 63x/1.40 oil DIC M27 objective or a Zeiss LSM510 equipped with a Plan-Apochromat 63x/1.40 oil phase contrast objective. Images were then analyzed with Image J (see supplemental Fig. S1 and S3).

Heatmap generation - The heatmap was built using directly FC values of selected proteins between 10 and 30 min (M/L), 30 and 60 min (H/M) and 10 and 60 min (H/L). Proteins have been clustered using a distance correlation and average linkage (see supplemental Fig. S7).

Supplemental Table legend

Supplemental Table S1. **SILAC ratios and gene ontology analysis of signaling endosome proteins.** This table includes all identified proteins, their ID and gene names, 30/10 (M/L), 60/10 (H/L) and 30/60 (H/M) ratios or fold-change, all provided by the MaxQuant/Andromeda software. Label-free intensity-based absolute quantification (iBAQ) values were calculated according to Schwanhausser et al (2013) (1). Proteins receiving higher iBAQ values from a GST-MIONs-carriers purification compared to the iBAQ values from a H_CT-MIONs-carriers purification, were categorized as '+' in the Tox<GST column, indicating that the corresponding protein is likely to be non-specific. Gene ontology analysis was provided by QuickGO. NaN (not a number) indicates that the corresponding proteins were identified but not quantified. PEP: posterior error probability.

Supplemental Table S2. **Specific signaling endosome proteins enriched at early time points of internalization.** This table summarizes all proteins whose 30/10 (M/L) and/or 60/10 (H/L) SILAC ratios or fold-change display a decrease during the experiment. The majority of these proteins are found in the zoom plot in Fig. S4. The columns are the same as described for Table S1, except that only signaling-endosome specific proteins are included.

Supplemental Table S3**. Specific signaling endosome proteins showing a temporal dependence during axonal transport and maturation.** This table summarizes all proteins whose 30/10 (M/L) and/or 60/10 (H/L) SILAC ratios display a temporal dependence during the experiment, according to the significance B score of their fold-change. The columns are the same as described for Table S1, except that only signaling-endosome specific proteins are included.

Supplemental Figure legends

Supplemental FIG. S1. **H_CT internalization in ES-derived motor neurons.** *A*, DIV3 ES-derived MNs were fixed and processed by immunofluorescence to detect the MN-specific markers Islet 1/2 (green) and ChAT (red), and the neuronal marker βIII-tubulin (blue). 20% of cells in this culture display MN markers. *B*, DIV3 ES-derived MNs were incubated with H_CT for 60 min at 37 °C, acid-washed, fixed, and processed by immunofluorescence to detect ChAT (green), HB9 (blue) and HA-H_CT (red). Binding and perinuclear accumulation of H_CT was only observed in MNs, as demonstrated by the lack of H_CT signal in glial cells (red asterisks). Nuclei in both experiments were stained with DAPI. Single plane images are shown. Scale bars: 5 μm.

Supplemental FIG. S2. **SILAC labeling incorporation in ES-derived MNs.** MaxQuant calculated peptide intensities for each isotopologue were summed and converted to percentages. Red and blue bars indicate the percentage of intensity associated with the labeled and unlabeled peptides, respectively. R: arginine, K: lysine, P: proline.

Supplemental FIG. S3. **H_CT-MIONs internalization in ES-derived motor neurons.** DIV3 ES-derived MNs were incubated with H_CT-MIONs for 60 min at 37 °C, acid-washed, fixed, and processed by immunofluorescence to detect HA-H_CT-MIONs (red), βIII-tubulin (green) and the MN-specific promoter HB9 (blue). Single plane images are shown. The inset shows the high magnification of a MN soma. Scale bars: 10 μm.

Supplemental FIG. S4. **Proteins enriched in the MS analysis at early time points of the internalization kinetics.** The bottom left quadrant of the SILAC FC scatter plot (Fig. 2*B*) was magnified in order to examine the components whose abundance decreased after 10 min of internalization. Proteins with the highest average change were annotated with the corresponding gene names. Only signaling endosome-specific proteins are displayed.

Supplemental FIG. S5. **Cell compartment distribution of signaling endosome components by Gene Ontology analysis.** QuickGO terms were overlaid onto the SILAC FC scatter plot, from which potential non specific proteins had been removed. *A,* Mitochondrion. *B,* Nucleus. The proteins of a given compartment are highlighted in purple and annotated with corresponding gene names.

Supplemental FIG. S6. **FC ratio significance.** Significance B was calculated as described by Cox and Mann (2008) (2). The cloud of data exhibits a lower spread at high protein abundance, indicating a more precise quantification. To capture this effect, outliers were defined for protein subsets obtained using intensity binning. The 30/10 (*A*) and 60/10 (*B*) FC ratios were plotted against corresponding log10-transformed protein intensities (sum of peptide intensities). The FC ratio data points were colored by their significance B score, with red circles having values <0.05. All significant B 30/10 and 60/10 FC ratios were plotted against each other on this new SILAC FC scatter plot (*C*). As described in Fig. 2*B*, proteins associated with high intensities were plotted with larger point sizes. Only significant B components were included in the temporal-dependent proteins pool.

Supplemental FIG. S7. **Trend signature of temporal-dependent signaling endosome proteins.** The heatmap shows the fold change (FC) values for the 262 selected proteins exhibiting significant time dependence. The columns correspond to the 30 min/10 min, 60 min/30 min and 60 min/10 min FC. The corresponding color bar legend is located on the top left of the heatmap.

REFERENCES

1. Schwanhausser, B., Busse, D., Li, N., Dittmar, G., Schuchhardt, J., Wolf, J., Chen, W., and Selbach, M. (2013) Corrigendum: Global quantification of mammalian gene expression control. *Nature* **495**, 126-127

2. Cox, J., and Mann, M. (2008) MaxQuant enables high peptide identification rates, individualized p.p.b.-range mass accuracies and proteome-wide protein quantification. *Nat Biotechnol* **26**, 1367-1372
